# Development of Low-Smoke Epoxy Resin Carbon Fiber Prepreg

**DOI:** 10.3390/polym17192710

**Published:** 2025-10-09

**Authors:** Yu Zhao, Lili Wu, Yujiao Xu, Dongfeng Cao, Yundong Ji

**Affiliations:** 1 School of Materials Science and Engineering, Wuhan University of Technology, Wuhan 430070, China; 15135955297@163.com (Y.Z.); polym_wl@whut.edu.cn (L.W.); 18339748046@163.com (Y.X.); 2 State Key Laboratory of Advanced Technology for Materials Synthesis and Processing, Wuhan University of Technology, Wuhan 430070, China; 3 Foshan Xianhu Laboratory of the Advanced Energy Science and Technology Guangdong Laboratory, Foshan 528000, China; 4 Institute of Advanced Materials and Manufacturing Technology, Wuhan University of Technology, Wuhan 430070, China

**Keywords:** epoxy resin, polysiloxane, modification, prepreg, low-smoke

## Abstract

The smoke toxicity of epoxy resin limits the application of its carbon fiber composites in marine interior structures. To address this issue, a novel epoxy resin (EZ) was synthesized by grafting phenyl propyl polysiloxane (PPPS) onto ortho-cresol novolac epoxy resin (EOCN), building upon the group’s earlier work on polysiloxane-modified epoxy resin (EB). The results confirmed successful grafting of PPPS onto EOCN, which significantly enhanced the thermal stability and char residue of EZ. Specifically, the peak heat release rate (PHRR), total heat release (THR), peak smoke production rate (PSPR), and total smoke production (TSP) of EZ were reduced by 68.5%, 35%, 73.1%, and 48.3%, respectively, attributable to the formation of a stable and compact char layer that suppressed smoke generation. By blending EZ with EB resin, a low-smoke epoxy system (LJF-2) was developed for prepreg applications. Carbon fiber composites (LJF-CF) prepared from LJF-2 exhibited minimal smoke emission and a unique bilayer char structure: a dense inner layer that hindered smoke transport and a thick outer layer that provided thermal insulation, delaying further resin decomposition. Silicon was uniformly distributed in the char residue as silicon oxides, improving its stability and compactness. Without adding any flame retardants or smoke suppressants, LJF-CF achieved a maximum smoke density (Ds,max) of 276.9, meeting the requirements of the FTP Code for ship deck materials (Ds,max < 400). These findings indicate that LJF-CF holds great promise for use in marine interior components where low smoke toxicity is critical.

## 1. Introduction

Carbon fiber-reinforced composites have been widely utilized in aerospace, rail transportation, marine engineering, and other fields due to their exceptional properties, including lightweight design, corrosion resistance, and fatigue resistance [1,2]. However, safety and environmental concerns, particularly smoke toxicity, have constrained their further application in critical areas such as marine interior components, where stringent smoke emission standards are mandated. The *International Maritime Organization* (*IMO*) explicitly stipulates in the *International Code for Application of Fire Test Procedures* (*FTP Code*) that the specific optical density of smoke (D_s_) for materials used in shipboard bulkheads must not exceed 200. Therefore, developing low-smoke carbon fiber composite systems through in-depth exploration of smoke suppression mechanisms holds significant scientific and engineering value, addressing both performance demands and regulatory compliance in high-risk environments.

The resin matrix is a critical factor influencing the smoke generation of carbon fiber-reinforced composites. Current methods to suppress smoke emission and enhance flame retardancy in resins and composites primarily involve the addition of silicon-, phosphorus-, and nitrogen-based flame retardants or inorganic fillers [3,4,5,6]. Further improvements in flame retardancy and smoke suppression can be achieved by modifying or functionalizing these additives (e.g., co-doping) to optimize their dispersion and reactivity within the resin matrix.

You et al. [7] synthesized a smoke suppressant (PNS) using 9,10-dihydro-9-oxa-10-phosphaphenanthrene-10-oxide (DOPO), benzaldehyde, and 4,4′-diaminodiphenyl sulfone (DDS) as raw materials. At a phosphorus content of 1.5 wt%, the PNS-modified resin exhibited a 15.4% reduction in total smoke production (TSP) compared to DOPO alone. Liu et al. [8] developed an active P/N/Si-containing flame retardant (KDC) via aldehyde-amine condensation. Cone calorimetry results showed that with 7 wt% KDC, the peak of smoke production rate (PSPR) and TSP of the epoxy resin decreased by 5% and 23.8%, respectively.

The protective effect of silicon oxide, the catalytic carbonization of phosphorus element and the quenching effect on reactive free radicals, and the dilution effect of non-combustible nitrogen oxides on combustible gases, all reduced the thermal decomposition rate and degree of the resin. In systems like PNS and KDC, which integrate Si, P and N, these mechanisms synergistically inhibit resin pyrolysis and combustion, achieving dual flame retardancy and smoke suppression. This multi-element approach highlights the potential of co-doping strategies to advance fire-safe composite design.

In addition to the aforementioned organic flame retardants, inorganic smoke suppressants such as aluminum hydroxide and graphene have also been extensively studied and applied. Chai et al. [9] found that doping epoxy resin with 15 wt% Al(OH)_3_ (ATH) reduced the TSP of the resin by 17.65% and decreased the carbon monoxide generation rate by 30.24%. This is attributed to the alumina film formed by Al(OH)_3_ at high temperatures, which adheres to the residual char of the resin, acting as a barrier to reduce heat and smoke exchange rates—a mechanism analogous to the role of silica produced by the thermal decomposition of polysiloxane. Xu et al. [10] prepared a Mg-Al-layered double hydroxide-supported graphene hybrid material (RGO-LDH) via a co-precipitation method, which reduced the total smoke yield of epoxy resin by 28.2%. Wang et al. [11] synthesized metal oxide-modified Co_3_O_4_-graphene and SnO_2_-graphene hybrid materials as smoke suppressants. Thermogravimetric Analysis—Fourier Transform Infrared Spectroscopy analysis (TG-FTIR) revealed that both hybrid materials decreased the absorbance of organic volatiles (carbonyl and aromatic compounds) in the resin, corresponding to a reduction in volatile organic content. Since these volatiles are the primary source of smoke particles [12], such graphene hybrid materials leverage the synergistic effects between the catalytic activity of metal oxides and the adsorption capacity of graphene to suppress smoke generation by minimizing organic volatiles.

Based on the aforementioned research, the current smoke suppression mechanisms of epoxy resins can be summarized as follows: (1) Dilution effect of inorganic fillers: The incorporation of inorganic fillers reduces the resin (combustible material) content. However, this approach requires high filler loading (e.g., 15 wt% ATH in the study) to achieve modest smoke suppression (e.g., <20% TSP reduction in epoxy resin). Excessive filler addition inevitably deteriorates the resin’s rheological properties, leading to processing challenges, and compromises mechanical performance. (2) Adsorption by graphene hybrid materials: Graphene-based hybrids mitigate smoke generation by adsorbing organic volatiles that evolve into smoke particles. The adsorption efficiency depends on the hybrid’s adsorption capacity and loading. Similar to fillers, excessive hybrid content negatively impacts resin processability and mechanical properties. (3) Intrinsic flame-retardant and smoke-suppressing behaviors of heteroatoms: Silicon: forms thermally stable silica layers upon pyrolysis, enhancing the barrier effect of residual char. Phosphorus: Promotes catalytic carbonization and releases phenoxy radicals to quench active free radicals in the gas phase. Nitrogen: Generates non-flammable gases during decomposition and stabilizes residual char. In co-doped systems, these effects synergize: Phosphorus and nitrogen primarily contribute to gas-phase and condensed-phase flame retardancy, while silicon, upon forming silicon oxides, enhances the stability and compactness of the char layer, thereby providing superior smoke suppression.

**Figure 1:**
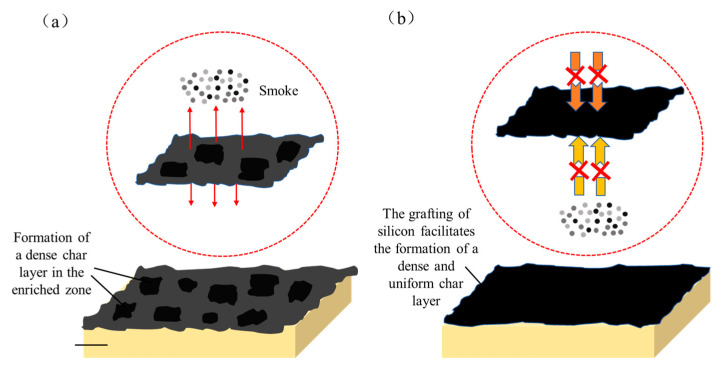
Effect of Char Layer Compactness on Smoke Generation: Additive flame retardants (**a**), Modified flame retardant (**b**). (The arrow indicates the direction of smoke propagation.).

Additive flame retardants often face challenges in achieving uniform dispersion within resins. Even reactive silicon-containing flame retardants may suffer from phase separation-induced aggregation, leading to similar inhomogeneity issues. Consequently, during combustion, the resin struggles to rapidly form a homogeneous char layer (as illustrated in Figure 1a), resulting in limited smoke suppression. In contrast, our group previously chemically modified a bisphenol A-based epoxy resin (E51) using phenyl propyl polysiloxane (PPPS) and 10-(2,5-dihydroxyphenyl)-10-hydro-9-oxa-10-phosphaphenanthrene-10-oxide (DOPO-HQ), yielding two modified resins designated as B-30 (silicon-modified) and P-27 (phosphorus-modified). These were then blended to produce a homogeneous B-P resin. Cone calorimetry tests revealed that the TSP of B-30, P-27, and B-P resins decreased by 54.8%, 16.3%, and 30.5%, respectively, compared to unmodified E51 [13].

This modification strategy introduces silicon and phosphorus into the epoxy’s molecular network via chemical grafting, ensuring their uniform distribution. Under high temperatures, silica derived from PPPS is evenly dispersed within the char residue (Figure 1b), forming a denser and more continuous protective layer. This structure effectively delays pyrolysis and significantly reduces smoke emission. However, the smoke suppression efficiency of the Si-P co-doped system was inferior to that of the silicon-only system (B-30), aligning with the earlier conclusion that silicon exhibits superior smoke-inhibiting effects. Therefore, this study exclusively employs silicon modification (via PPPS) for epoxy resin optimization.

Prepreg-based composite manufacturing offers significant advantages, including ease of processing, low porosity, controllable curing, and exceptional mechanical properties, making it a dominant process for high-performance composites. However, in previous work, the PPPS-modified E51 resin didn’t fabricate directly prepreg due to its low viscoelasticity. To address this challenge, this study proposes a low-smoke Ortho-Cresol Novolac Epoxy Resin (EOCN) system based on PPPS-modified E51. The newly designed resin is a solid-state linear multifunctional epoxy characterized by enhanced thermal stability and mechanical robustness. By strategically blending solid and liquid epoxy resins, the rheological properties of the hybrid system were optimized to meet prepreg processing requirements. Both modified resins incorporate silicon via chemical grafting, achieving superior elemental distribution compared to physically blended additives. This homogeneous dispersion facilitates the formation of a continuous silica-rich char layer during combustion, significantly improving smoke suppression efficiency.

This study employs PPPS as a modifier to chemically functionalize EOCN and E51. The structural modifications were characterized using Fourier-transform infrared spectroscopy (FTIR), and the smoke emission of both resins before and after modification was evaluated via cone calorimetry. Subsequently, the two modified resins were blended at varying mass ratios to prepare hybrid resin systems. The rheological properties of these hybrids were measured using a cone-plate viscometer to identify an optimal formulation meeting prepreg processing requirements. Following this, modified resin-based composites were fabricated using the optimized formulation. Their smoke generation behavior was systematically investigated through cone calorimetry and single-chamber smoke density tests, providing comparative insights into the efficacy of the silicon-modified resin system.

## 2. Experiment

### 2.1. Materials

The materials used in this study include: Bisphenol A epoxy resin (E51) and ortho-cresol novolac epoxy resin (EOCN), purchased from Sinopec Hunan Petrochemical Co., Ltd. (Yueyang, China); phenyl propyl polysiloxane (PPPS, industrial grade), supplied by Dow Corning Corporation (Midland, MI, USA); Dibutyltindilaurate (DBDTL, 95.0%) and acetone (analytical grade), both obtained from Sinopharm Chemical Reagent Co., Ltd. (Shanghai, China); Dicyandiamide (DICY, analytical grade), sourced from Tianjin Huasheng Chemical Reagent Co., Ltd. (Tianjing, China); Unidirectional carbon fiber fabric (T700-12K), procured from Zhongfu Shenying Co., Ltd. (Lianyungang, China).

### 2.2. Synthesis of Modified Resins

#### 2.2.1. Synthesis of PPPS-Modified E51

The synthesis method and procedures for PPPS-modified E51 followed the protocols detailed in the group’s prior publications [13]. The resulting modified resin was designated as EB.

#### 2.2.2. Synthesis of PPPS-Modified EOCN

Predetermined mass ratios of EOCN and PPPS were weighed and transferred into a three-neck flask. The mixture was heated to a certain temperature, followed by the addition of acetone (solvent) to fully dissolve the reactants. A controlled amount of DBDTL catalyst was introduced into the flask. The reaction proceeded at 50 °C for 3 h under continuous stirring, yielding a white semi-transparent intermediate. The crude product was placed in a vacuum oven and dried under reduced pressure at 100 °C to evaporate residual acetone, resulting in a pale-yellow PPPS-modified EOCN (EZ).

### 2.3. Sample Preparation

#### 2.3.1. Preparation of Resin Castings

Based on the measured epoxy value of the resin, the stoichiometric amount of DICY curing agent was calculated to ensure complete crosslinking. The resin and DICY were mechanically mixed until homogeneous, followed by degassing in a vacuum oven at 60 °C for 30 min to eliminate trapped air bubbles, which could otherwise lead to void formation and compromised mechanical properties during curing. The degassed mixture was then poured into preheated molds and subjected to a stepwise curing protocol in a forced-air convection oven.

#### 2.3.2. Cone-and-Plate Viscometry

The EZ and EB resins were blended at mass ratios of 7:3, 6:4, 6.5:3.5, 5:5, and 4:6 (EZ:EB), with a fixed loading of curing agent added to each mixture. The resulting hybrid resins were designated as LJF-1, LJF-2, LJF-3, LJF-4, and LJF-5, respectively. Cone-and-plate viscometry was employed to evaluate the viscosity-temperature profiles of hybrid resin formulations, identifying the optimal composition for prepreg processing based on rheological performance.

#### 2.3.3. Preparation of Prepreg

**Figure 2:**
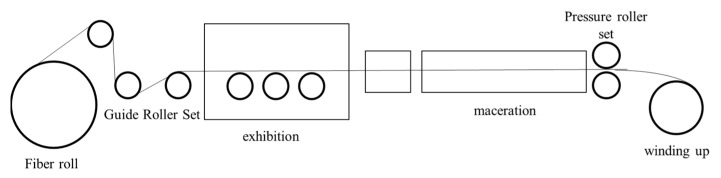
Flowchart of Carbon Fiber Prepreg Manufacturing Process.

**Figure 3:**
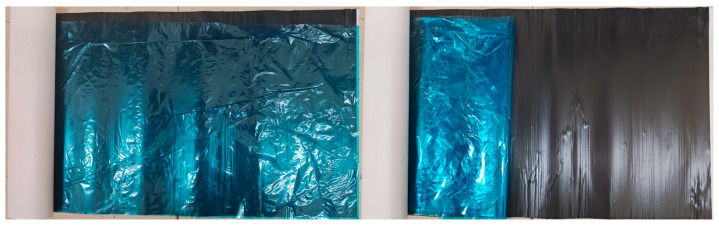
Macro Image of Prepreg Sample.

The hybrid resin formulation optimized via cone-and-plate viscometry (Section 2.3.2) was selected as the matrix resin, combined with T700-12K unidirectional carbon fiber fabric as the reinforcement, to fabricate prepreg using a continuous prepreg production line. Figure 2 illustrates the prepreg manufacturing process, including critical steps such as resin impregnation, fiber spreading, and composite compaction, while the macroscopic morphology of the resulting prepreg is shown in Figure 3.

#### 2.3.4. Preparation of Composite Material

**Figure 4:**
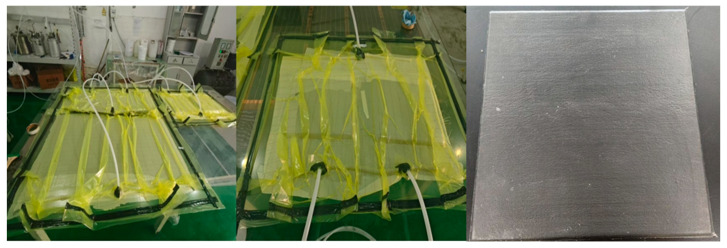
Preparation and Physical picture of LJF-CF composite material.

The prepared prepreg was cut to the specified dimensions. Based on the thickness requirement for cone calorimetry test samples (3.0 ± 0.2 mm) per ISO 5660-1 standard [14], 12 plies were determined as the optimal layup configuration. A unidirectional lay-up process was employed for stacking, and the layered structure was then placed in a preheated mold. The compression molding process was conducted using the following curing protocol: 100 °C/2 h + 150 °C/2.5 h + 160 °C/3 h, A constant pressure of 5 ± 0.2 MPa was maintained throughout the curing process. The resulting carbon fiber-reinforced composite material was designated as LJF-CF (Figure 4).

**Figure 5:**
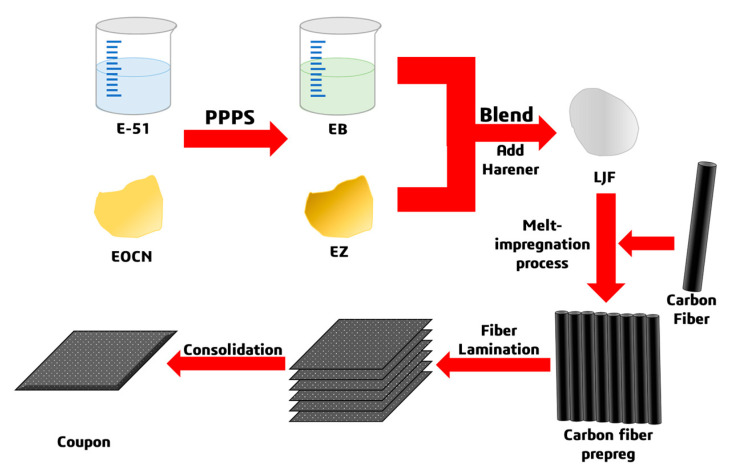
Experimental procedure flowchart.

Carbon fiber composites, named E51-CF and EB-CF, were prepared by vacuum bagging and pressing method using E51 and EB resins as matrix, respectively. Carbon fiber composites, named EZ-CF, were prepared by hand-lay-up molding using EZ resin as matrix(The sample preparation process is shown in Figure 5). 

### 2.4. Characterization

(1) Infrared testing: Infrared spectroscopy of EOCN, PPPS, and EZ was carried out by KBr compression method using a Nicolet 6700 FTIR spectrometer (Waltham, MA, USA) with a scanning wave number range of 4000–5000 cm^−1^;
(2) Thermogravimetric analysis was performed using a STA449F3 simultaneous thermal analyzer (Selb, Germany). The resin sample was heated from 40 to 1000 °C at a constant heating rate of 10 °C/min under a dynamic air atmosphere.
(3) Cone Calorimetry Test: According to ISO 5660 standard, FTT 0007 Cone Calorimeter is used to conduct cone calorimetry test on resin and composite materials.
(4) The microscopic morphology of char residues of the resin and the composites was observed, and spot elemental analysis was performed using an FEI Scios 2 scanning electron microscope (SEM, Hillsboro, OR, USA) equipped with energy-dispersive X-ray spectroscopy (EDS, Hillsboro, OR, USA) at an accelerating voltage of 20 kV. Prior to analysis, the sample surfaces were sputter-coated with platinum to enhance conductivity.
(5) Smoke Density Testing: In accordance with ISO 5659-2 [15], a single-chamber smoke density test was performed on the composite material to determine the specific optical density of smoke (abbreviated as smoke density). This test was performed to assess compliance with the smoke toxicity requirements of the *International Maritime Organization*’s FTP Code.

## 3. Results and Discussion

### 3.1. Structure and Properties of the Matrix Resin

#### 3.1.1. Structural Characterization

**Figure 6:**
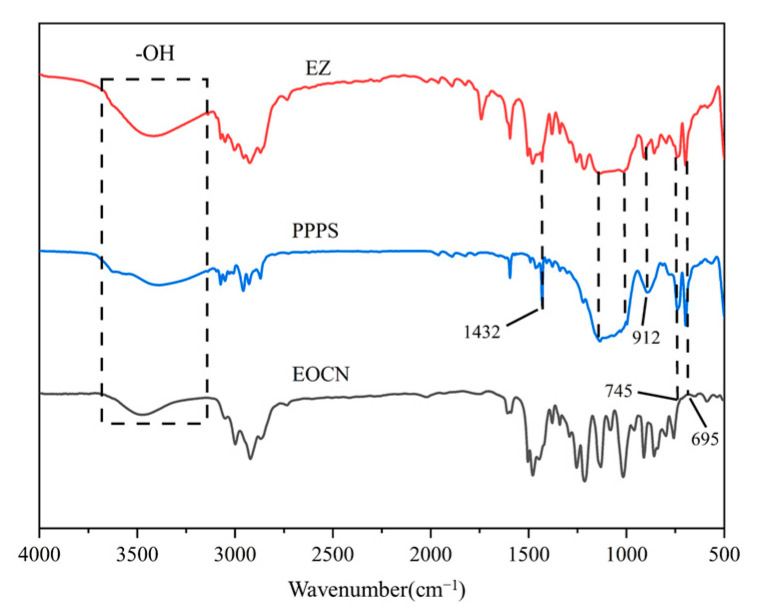
Infrared spectra of EOCN, EZ, and PPPS.

The FTIR spectra of EOCN, EZ resin, and PPPS are shown in Figure 6. In the spectrum of EOCN, the characteristic peak at 3481 cm^−1^ corresponds to the O–H stretching vibration of hydroxyl groups, while the peak at 912 cm^−1^ is attributed to the stretching vibration of epoxy groups [16]. For PPPS, distinct absorption bands are observed at:1432 cm^−1^: Si–C_6_H_5_ (phenyl-silicon bond) stretching, 745–695 cm^−1^: Si–C_3_H_7_ (propyl-silicon bond) bending and 1200–1000 cm^−1^: Si–O–C (siloxane-ether linkage) asymmetric stretching. In the FTIR spectrum of EZ resin, all characteristic peaks of PPPS are retained, while the intensity of the epoxy group peak at 912 cm^−1^ is significantly reduced, indicating partial consumption of epoxy groups during the reaction between EOCN and PPPS. Additionally, compared to EOCN, the O–H stretching vibration peak in EZ resin broadens noticeably around 3450 cm^−1^, which arises from hydrogen bonding interactions between the Si–OH terminal groups of PPPS and the epoxy groups of EOCN. These phenomena confirm the successful chemical grafting of PPPS onto the EOCN backbone.

The structural characterization of EB resin is consistent with prior work by the research team and is not discussed here [17].

#### 3.1.2. Heat Resistance Analysis

**Figure 7:**
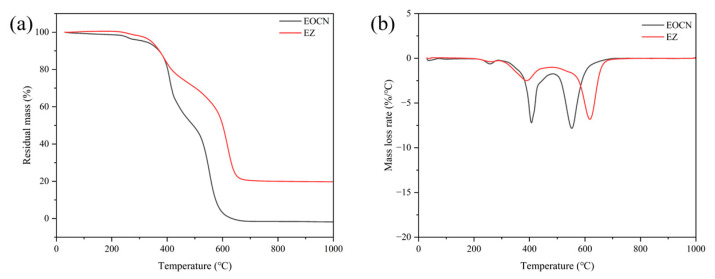
TG (**a**) and DTG (**b**) curves of EOCN and EZ resin.

The thermogravimetric data of EOCN and EZ resins are summarized in Table 1, with corresponding TG-DTG curves shown in Figure 7. The temperature at 5% mass loss (T_5%_) for EOCN and EZ resins was 318.2 °C and 342.8 °C, respectively. Compared to EOCN, the T_5%_ of EZ resin increased by 7.7%, indicating that the introduction of PPPS delayed the pyrolysis of EOCN. The first-stage maximum decomposition rate temperature (T_max1_) for EOCN and EZ resins was 407.1 °C and 390.1 °C respectively, while the second-stage T_max2_ was 552.5 °C and 617.5 °C. Notably, the maximum mass loss rate (MLR_max_) of EZ resin (6.8%/°C) was lower than that of EOCN (7.8%/°C). The EZ resin exhibited a 17 °C reduction in T_max1_ compared to EOCN but demonstrated a significantly slower decomposition rate. EZ resin showed enhanced thermal stability, with T_max2_ increased by 65 °C relative to EOCN. PPPS improves the thermal stability of EOCN by delaying pyrolysis and reducing the maximum decomposition rate.

The introduction of PPPS into EOCN resin introduced high-bond-energy Si–O bonds (bond energy: 452 kJ/mol) and Si–C bonds (bond energy: 318 kJ/mol). The cleavage of these bonds during thermal decomposition requires additional energy absorption, thereby elevating the material’s decomposition temperature [18]. Under air atmosphere at 800 °C, the char residue of EOCN and EZ resins was 0% and 20%, respectively. The incorporation of PPPS significantly enhanced the char-forming capability of the matrix resin, which directly contributes to its smoke suppression efficacy in subsequent combustion tests.

The oxidative degradation of PPPS proceeds via a free radical reaction mechanism. During the initial degradation stage, side groups on the PPPS molecular chain form peroxide intermediates, which subsequently undergo β-scission reactions, releasing volatile small molecules. As degradation progresses, the siloxane backbone gradually transforms into thermally stable silica (SiO_2_) [19,20]. The resulting SiO_2_ is uniformly dispersed within the char layer, creating a dense barrier that impedes oxygen diffusion and suppresses heat exchange, thereby delaying the decomposition of the underlying resin matrix and significantly enhancing the thermal stability of the EZ resin. The thermal resistance of EB resin has been extensively characterized in prior work by the research team and is not discussed here [17].

#### 3.1.3. Cone Calorimetric Analysis

**Figure 8:**
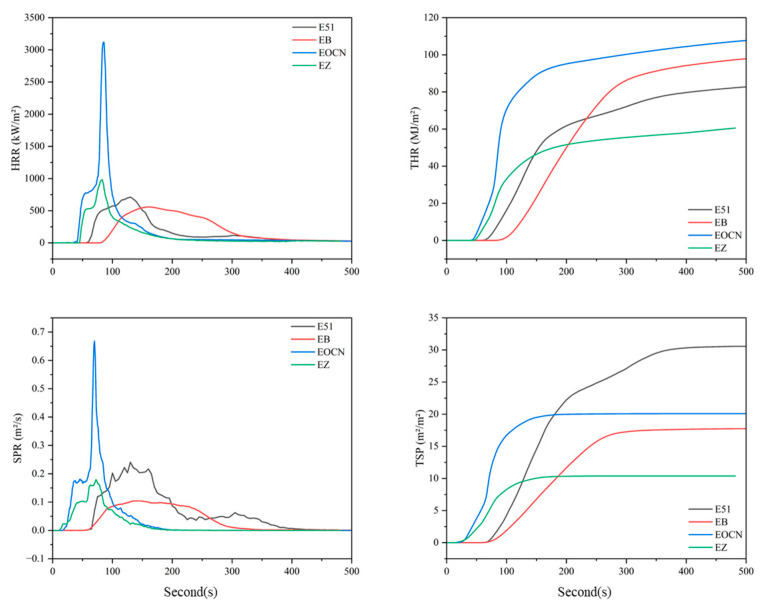
Cone Calorimetry Curves of E51, EB, EOCN and EZ Resins.

Cone calorimetry tests were performed on resin castings of E51, EB, EOCN, and EZ. The combustion data were compiled in Table 2, with the corresponding curves presented in Figure 8.

As shown in Table 2, the peak of heat release rate (PHRR) of E51, EB, EOCN, and EZ resins are 710.9 kW/m^2^, 672.5 kW/m^2^, 3121.4 kW/m^2^, and 981.87 kW/m^2^, respectively. Compared to their unmodified counterparts, the PHRR of EB and EZ resins decreased by 5.4% and 68.5%, respectively. The total heat release (THR) of E51 and EB resins showed no significant difference, while the THR of EZ resin was 35% lower than that of EOCN. PHRR and THR reflect the intensity of combustion and are critical parameters for assessing fire hazards [20]. The substantial reduction in both HRR and THR for EZ resin demonstrates its intrinsic flame retardancy, attributable to the incorporation of PPPS.

This performance enhancement is attributed to the high bond energy of Si–O bonds in PPPS, which require greater energy absorption during bond cleavage, thereby delaying the pyrolysis of the material. As demonstrated in prior thermal analysis, the oxidative decomposition of PPPS generates thermally stable silica (SiO_2_). Crucially, the silicon is chemically grafted into the resin’s molecular network, and the resulting SiO_2_ is uniformly distributed within the char residue, forming a dense and stable protective layer. This layer effectively blocks oxygen diffusion and retards the release of flammable pyrolysis gases, suppresses heat exchange between the combustion zone and the underlying resin matrix, thereby inhibiting further pyrolysis of the inner resin layers [21].

Compared to their unmodified counterparts, the PSPR of EB and EZ resins decreased by 58.3% and 73.1%, respectively, while their total TSP was reduced by 54.8% and 48.3%. The silica-rich protective char layer not only blocks heat transfer and delays resin degradation but also suppresses the majority of smoke particle emissions.

As evidenced by thermal analysis, PPPS significantly enhances char residue formation. The increased char residue content in EB and EZ resins effectively traps organic volatiles (primary precursors of smoke particles), leading to marked smoke reduction. Under continuous thermal radiation, pyrolysis products accumulate internally, generating pressure that induces microcracks in the char layer. These microcracks temporarily enhance gas/heat exchange, intensifying combustion. Subsequently, newly formed pyrolysis products deposit on the surface, repairing the cracks and regenerating a dense char layer. This self-repairing mechanism drives a cyclic densification-cracking process, explaining the fluctuating (non-smooth) SPR curves.

The substantial reductions in SPR and TSP demonstrate that PPPS grafting imparts intrinsic flame-retardant and smoke-suppressive properties to the epoxy resin matrix. This chemical modification strategy outperforms conventional additive-type flame retardants/smoke suppressants, which suffer from inhomogeneous dispersion and leaching risks.

#### 3.1.4. Char Residue Analysis of Resin Matrix Combustion

**Figure 9:**
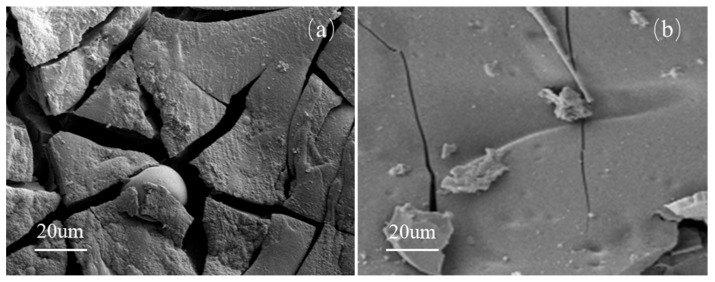
Microscopic morphology of char residues from (**a**) EOCN and (**b**) EZ resins.

Figure 9 illustrates the microscopic morphology of the char residues from EOCN and EZ resins, with the elemental composition summarized in Table 3. As shown in the images, the char residue of EOCN (Figure 9a) exhibits a smooth surface traversed by large-width cracks, which serve as channels for heat transfer and smoke transport, accelerating resin pyrolysis. This explains the higher heat release and smoke production observed for EOCN in cone calorimetry tests. In contrast, the char residue of EZ resin (Figure 9b) is uniform and dense, with only minor microcracks on the surface. This compact structure effectively isolates heat and smoke transfer, suppressing the pyrolysis of the underlying resin matrix. The microscopic morphology of the residual carbon of these two resins explains well the results of the cone calorimetry tests.

The incorporation of PPPS into the EOCN molecular network promotes the formation of stable silica oxides during pyrolysis. These oxides enhance the thermal stability and structural compactness of the char residue, thereby achieving superior smoke suppression. The char residue analysis of E51 and EB resins has been detailed in prior work by the research group and is not reiterated here [13].

#### 3.1.5. Rheological Analysis

**Figure 10:**
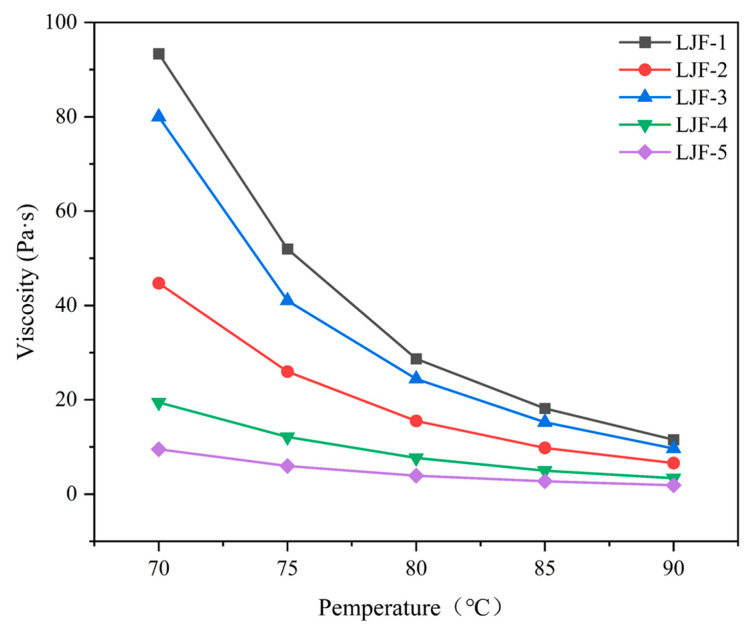
Viscosity-Temperature Profiles of Hybrid Resin.

The aforementioned cone calorimetry results demonstrate that PPPS significantly enhances the flame retardancy and smoke suppression performance of EOCN resin. Building on this achievement, to further optimize the processability of the resin, PPPS-modified EOCN resin (EZ) was blended with EB resin at varying mass ratios. The viscosity of the hybrid resins was systematically adjusted by tuning the EZ/EB ratio and evaluated at different temperatures. The viscosity-temperature profiles of these formulations are presented in the following sections (Figure 10).

Prepreg manufacturing imposes stringent requirements on resin viscosity. Within the optimal processing temperature range (80–90 °C), the matrix resin viscosity must be maintained within 10–20 Pa·s to ensure complete resin impregnation into fibers. Deviations from this range lead to critical issues: high viscosity (>20 Pa·s) leads to poor resin flow which results in incomplete fiber wetting; low viscosity (<10 Pa·s) leads to excessive resin drainage during prolonged gelation which causes localized resin-deficient zones in the prepreg [22].

As shown in Figure 10, the LJF-2 formulation (EZ:EB = 6:4) exhibits a viscosity of 10–20 Pa·s within the 80–90 °C prepreg processing window, fully meeting industrial requirements. This system offers two key advantages: optimal flow characteristics ensure rapid and uniform fiber impregnation, while controlled gel kinetics balance gel time to prevent resin bleed-out while maintaining processing efficiency. Notably, the viscosity of LJF-2 is consistently lower than that of LJF-3 due to the distinct morphologies of the liquid EB resin and the solid EZ resin. In LJF-3, the high-melting EZ particles are not fully plasticized, resulting in higher viscosity. In contrast, the 40% liquid EB content in LJF-2 acts as an effective plasticizer, enhancing chain mobility and reducing viscosity, thereby placing it perfectly within the target processing window for prepreg and demonstrating excellent flow behavior.

Based on this analysis, LJF-2 was selected as the matrix resin for prepreg production. Carbon fiber composites fabricated with this formulation were subsequently subjected to smoke density testing to validate its fire-safety performance.

### 3.2. Smoke Production Analysis of Composite Materials

#### 3.2.1. Cone Calorimetry Analysis

**Figure 11:**
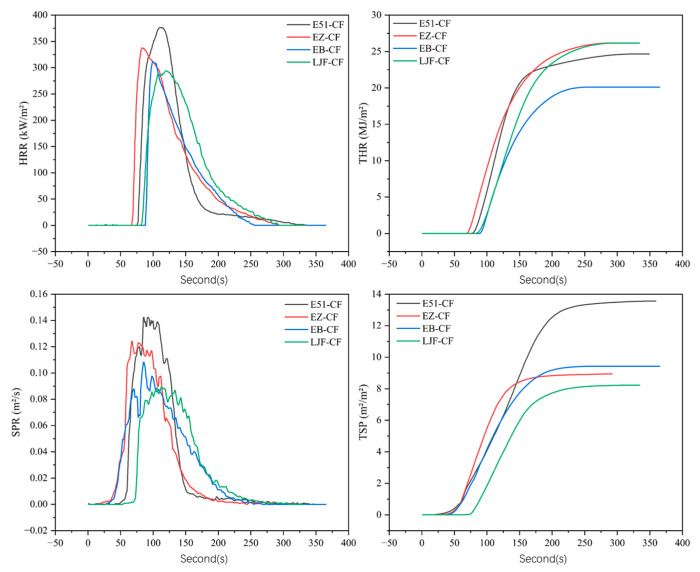
Cone calorimetric curves for composites.

Figure 11 presents the cone calorimetry results of composites with different resin matrices, including key combustion performance parameters such as HRR, THR, SPR, and TSP. The corresponding quantitative data are compiled in Table 4. The four composites exhibit three distinct HRR curve patterns: HRR curve of E51-CF shows a unique plateau-sharp decline characteristic, a brief deceleration in the rate of increase precedes the peak, followed by an abrupt drop, which means poor stability of the char layer formed by E51 resin results in weak and easily disrupted barrier properties, leading to rapid resin burnout. HRR curves of EB-CF and EB-CF display a rapid rise-gradual decline pattern, reaching peak values quickly and then decreasing slowly, indicating that the incorporation of PPPS significantly improves char quality. The silica-rich char layer exhibits enhanced stability and compactness, effectively reducing the pyrolysis rate of the underlying resin and prolonging flame-retardant efficacy. HRR behavior of LJF-CF resembles EB-CF/EZ-CF, but its SPR curve exhibits pronounced oscillations, which come from the dense-fracture dynamic cycling of the char layer: Char layer suppresses combustion, but crack formation intensifies combustion transiently. Alternation between these states creates fluctuating SPR profiles.

Under radiant heat, the resin matrix undergoes thermal expansion and melting, followed by pyrolysis and combustion. The carbon fiber bundle architecture, restrain resin melt flow and splashing, guide ordered accumulation of combustion products on the surface, form a continuous protective char layer. The in situ-formed char layer acts as an effective physical barrier by blocking heat transfer and oxygen diffusion, Significantly inhibiting further combustion of the underlying resin.

Compared to E51-CF, the PHRR of EB-CF, EZ-CF, and LJF-CF composites decreased by 17.2%, 10.5%, and 21.9%, respectively, while their PSPR was reduced by 21.4%, 14.3%, and 35.7%. Notably, LJF-CF exhibited the lowest SPR and TSP values, demonstrating the most effective smoke suppression performance. The smoke suppression mechanisms of composites are intrinsically linked to the structure of the char layer: Carbonaceous products formed during the initial combustion stage act as an effective physical barrier, significantly reducing heat transfer and gas exchange efficiency. This suppresses the pyrolysis kinetics of the underlying resin matrix; then the dense char layer physically blocks the release of organic volatiles and smoke particles generated during pyrolysis. The insulating effect of the char layer alters the chemical environment of the internal resin pyrolysis, potentially inducing alternative decomposition pathways that reduce the generation of volatile products.

In modified resins, silicon is homogeneously distributed within the polymer network. During combustion, the resulting silica (SiO_2_) uniformly disperses within the char layer, enhancing its stability by filling microcracks and pores. This structural reinforcement amplifies smoke suppression efficacy through these three synergistic mechanisms.

#### 3.2.2. Char Residue Analysis of Composite Materials

**Figure 12:**
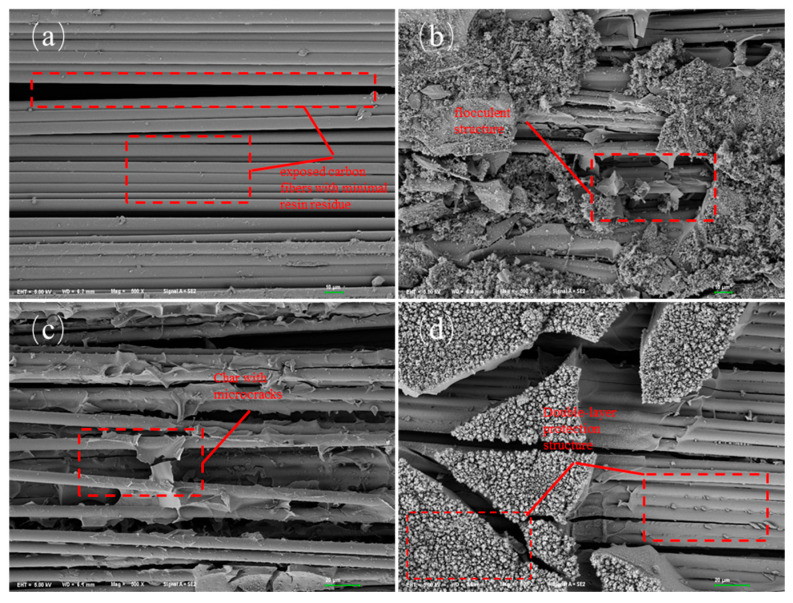
Microscopic morphology of char residue for composites: (**a**) E51-CF, (**b**) EB-CF, (**c**) EZ-CF, (**d**) LJF-CF.

Figure 12 illustrates the microscopic morphology of the char residue surfaces from four composite materials after cone calorimetry testing. The char residue of E51-CF (Figure 12a) is dominated by exposed carbon fibers with minimal resin residue. Fiber bundles appear dispersed, indicating complete resin burnout and the absence of a smoke-suppressive layer, which explains its high smoke production. The char residue of EB-CF (Figure 12b) exhibits a flocculent structure unevenly deposited on the fiber surface. Although a protective layer forms, its loose and discontinuous structure is prone to collapse under thermal flux, accelerating heat and smoke transfer. The char residue of EZ-CF (Figure 12c) shows improved fiber coverage, but numerous microcracks are distributed across the surface. These structural defects act as fast channels for heat transfer and smoke diffusion, compromising barrier efficacy.

The char residue of LJF-CF (Figure 12d) demonstrates a unique dual-layer protective architecture: Inner layer tightly coats the fibers with uniform and dense char, forming an effective smoke barrier; Outer layer accumulates atop the inner layer as additional char, creating a thermally insulating zone. This dual protection mechanism results in the lowest SPR and TSP values among all composites.

**Figure 13:**
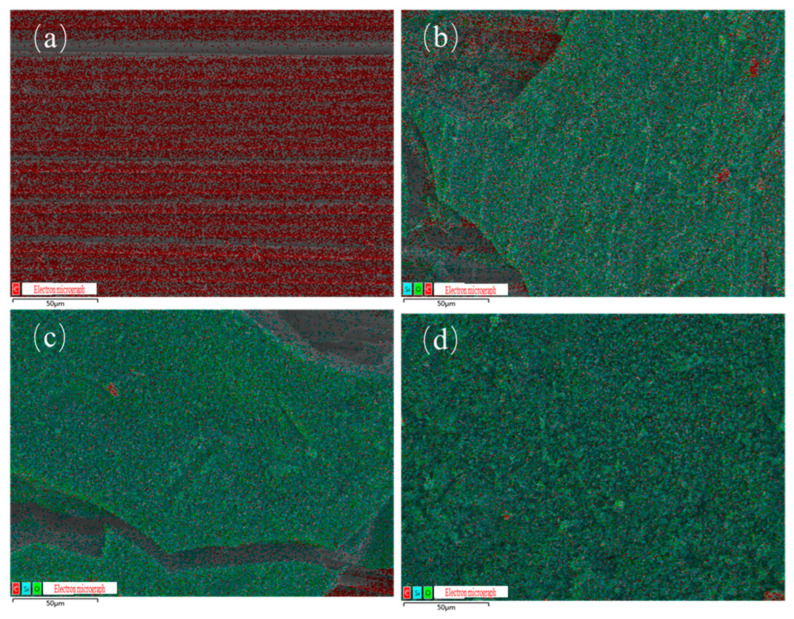
Elemental distribution of residue char for composites: (**a**) E51-CF, (**b**) EB-CF, (**c**) EZ-CF, (**d**) LJF-CF.

Figure 13a–d displays the elemental distribution maps of char residues for E51-CF, EB-CF, EZ-CF, and LJF-CF, with corresponding atomic percentages summarized in Table 5.

The char residue of E51-CF primarily contains carbon (C) from exposed carbon fibers, with negligible resin-derived elements. This aligns with its morphological features: complete resin burnout leaves fibers directly exposed to the environment.

The char residue of EB-CF (Figure 13b), EZ-CF (Figure 13c), and LJF-CF (Figure 13d) demonstrate uniform distribution of C, O, and Si, confirming the successful incorporation of PPPS into the resin matrix. Despite PPPS modification, carbon signals from underlying fibers are detected in EDS due to char layer cracks in the char residue of EB-CF and EZ-CF. In contrast, the char residue of LJF-CF exhibits a unique dual-layer structure: dense char fully encapsulates the fibers as inner layer; Outer layer enhances insulation as continuous thermal barrier. This structure effectively blocks the fiber signals detected by EDS. By combining the smoke production performance data and the analysis of the residue morphology, it can be concluded that by chemically grafting PPPS onto the molecular network of the epoxy resin, the silicon element can be uniformly distributed in the residue during combustion, improving the resin’s residue rate, as well as the thermal stability and compactness of the residue layer, thereby endowing the resin with excellent smoke suppression performance.

#### 3.2.3. Smoke Density Test

**Figure 14:**
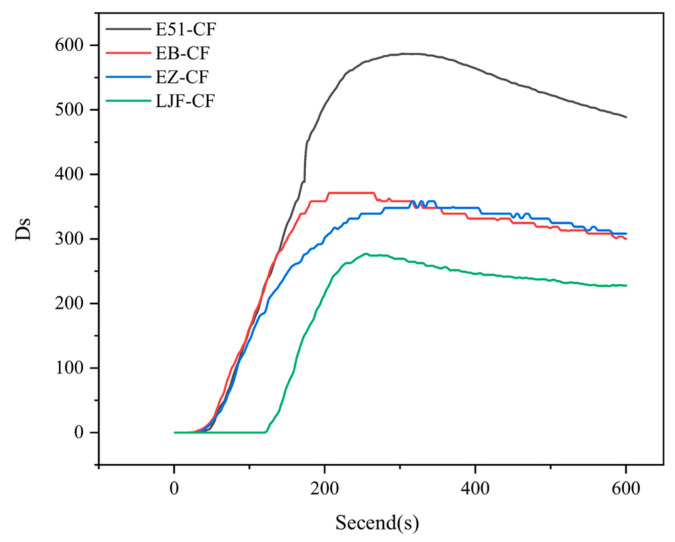
Smoke density profiles of composites.

To further validate the smoke suppression performance of the composites, single-chamber smoke density testing (ISO 5659-2) was conducted under controlled flaming conditions. Figure 14 illustrates the specific optical density of smoke (Ds) versus time curves for different materials, with the corresponding maximum smoke density values (Ds max) compiled in Table 6.

The single-chamber smoke density test quantifies real-time smoke density by measuring the attenuation of light transmission through smoke. As shown in Figure 13, during the 600-s test period, E51-CF exhibited a maximum smoke density (D_s_ max) near 600, attributed to intense smoke particle generation from uncontrolled resin pyrolysis and combustion due to the absence of flame-retardant/smoke-suppressive additives. EB-CF and EZ-CF achieved significantly lower D_s_ max values (~350), representing a ~50% reduction compared to E51-CF. There are two mechanisms by which smoke density decreases after the peak: First, the modified resin forms a silicon-enriched char layer that impedes heat transfer and smoke diffusion, suppressing continuous smoke particle generation. Second, gravitational settling of existing smoke particles increases light transmittance within the chamber over time. Among them, due to the lack of effective carbon layer protection, the smoke density value of E51-CF has always remained at a relatively high level. This result is highly consistent with the conical calorimetric test data, fully confirming that the introduction of PPPS endows the modified resin and its composite materials with obvious intrinsic flame retardant and smoke suppression properties. The practical application of PPPS modified resin in composite materials is feasible.

The maximum smoke density of LJF-CF is 276.9, which meets the *FTP Code* requirements for shipboard materials such as flooring and deck coverings (D_s_ max < 400), demonstrating its optimal smoke suppression performance. Based on these results, through further optimization of resin formulation (Adjusting PPPS content), manufacturing process (Refining curing protocols) and laminate design (Implementing hybrid layups), this prepreg system holds significant potential for development into high-performance and low-smoke composites compliant with maritime interior component standards.

## 4. Conclusions

In this study, PPPS was employed as a modifying agent to synthesize two modified epoxy resins, EB and EZ. When these two resins were compounded to prepare carbon fiber prepregs and composite materials, the following conclusions were drawn:

1. PPPS was chemically grafted into the molecular structures of E51 and EOCN resins, significantly improving their thermal stability and char residue yield. During combustion, silicon is uniformly distributed within the char residue as silicon oxides, forming a dense and homogeneous char layer that suppresses resin combustion, smoke transport, and reduces heat release and smoke generation. Compared to unmodified resins, the TSP of EB and EZ decreased by 54.8% and 48.3%, respectively. Compared with additive modification, the main reason for the significant smoke suppression effect is that the distribution of silicon elements introduced by grafting is more uniform, resulting in a more uniform distribution of silicon oxides after the resin pyrolysis.
2. The significant reduction in TSP of modified resin-based composites is primarily attributed to the structural characteristics of the silicon-containing char residue. Microstructural analysis reveals that the silicon-rich char residue generated from the pyrolysis of modified resins uniformly accumulates on the fiber surface, forming a continuous protective layer. Notably, the char residue of LJF-CF exhibits a dual-layer architecture: a dense inner layer acts as an effective smoke barrier, drastically reducing the diffusion of smoke particles; a thickened outer layer effectively insulated heat, and delays the pyrolysis kinetics of the underlying resin matrix. This “dual-layer protection” mechanism achieves smoke suppression through a synergistic effect.
3. LJF-CF composites meet the smoke density requirements for ship deck materials under the *FTP Code*. The LJF-CF composite material is a unidirectional carbon fiber laminatd unidirectionally structure, which only modify the resin matrix without adding any flame retardant/smoke suppressant. It is expected that through further smoke suppression measures, LJF-CF composites can be applied to the interior structural components of ships and other composite material fields with higher requirements for smoke toxicity.
4. Although chemical modification achieves superior smoke suppression and flame retardancy, it is more complex and costly than additive blending. This study successfully tuned the hybrid resin’s viscosity via formulation optimization to meet the prepreg processing window (10–20 Pa·s), acknowledging that not all EB/EZ resin ratios yield suitable rheology. Future work will focus on industrial scale-up, cost–benefit analysis, and assessing long-term durability in marine environments (aging, water/salt spray resistance). Additionally, mechanical property evaluation (tensile strength, flexural modulus, fracture toughness) will be expanded to validate structural performance.
5. The low-smoke prepreg developed in this study enriches the manufacturing techniques for low-smoke composites and significantly broadens their application scope.

## Figures and Tables

**Table 1:** Thermogravimetric data of EOCN and EZ resins.

Sample	T_5%_/°C	T_max1_/°C	T_max2_/°C	MLR_max_ (%/°C)	R_800_/%
EOCN	318.2	407.1	552.5	7.8	0
EZ	342.8	390.1	617.5	6.8	20

Note: T_5%_: Temperature at 5% mass loss; T_max1_ and T_max2_: Temperatures corresponding to the maximum mass loss rates in the first and second decomposition stages, respectively; MLR_max_: Maximum mass loss rate; R_800_: Char residue remaining at 800 °C.

**Table 2:** Cone calorimetric data for E51, EB, EOCN, EZ resins.

Sample	E51	EB	EOCN	EZ
PHRR (kW/m^2^)	710.9	672.5	3121.4	981.87
THR (MJ/m^2^)	80.8	83.3	85.46	55.56
PSPR (m^2^/s)	0.24	0.10	0.67	0.18
TSP (m^2^/m^2^)	30.5	13.8	20.08	10.39

Note: PHRR and PSPR are the peak of heat release rate and smoke production rate, respectively; THR and TSP are the total heat release and total smoke production, respectively.

**Table 3:** Percentage of residual carbon elements in the Resin Matrix.

Sample	Percentage of Residual Elements			
	C/wt%	O/wt%	Si/wt%	P/wt%
EOCN	74.7	25.0	0.2	0.1
EZ	56.6	22.3	21.0	0.1

**Table 4:** Cone calorimetric data for composites.

Sample	E51-CF	EB-CF	EZ-CF	LJF-CF
PHRR (kW/m^2^)	376.6	311.7	337.1	294.0
THR (MJ/m^2^)	24.67	20.12	26.19	26.16
PSPR (m^2^/s)	0.14	0.11	0.12	0.09
TSP (m^2^/m^2^)	13.5	9.4	9.0	8.2

**Table 5:** Percentage of residual carbon elements of Composite Materials.

Sample	Percentage of Residual Carbon Elements (%)		
	C	O	Si
E51-CF	100.00	0	0
EB-CF	50.06	37.94	12.00
EZ-CF	61.85	31.27	6.88
LJF-CF	59.08	30.84	10.08

**Table 6:** Maximum smoke density of composites.

Sample	D_s_ max
E51-CF	586.9
EB-CF	371.3
EZ-CF	358.5
LJF-CF	276.9

## Data Availability

The original contributions presented in this study are included in the article. Further inquiries can be directed to the corresponding author(s).
